# Higher prevalence of smoking and lower BMI, waist circumference, cholesterol and triacylglyceride levels in Prague's homeless compared to a majority of the Czech population

**DOI:** 10.1186/1471-2458-7-51

**Published:** 2007-04-05

**Authors:** Dana Kubisová, Věra Adámková, Věra Lánská, Pavel Dlouhý, Jolana Rambousková, Michal Anděl

**Affiliations:** 1 Department of Nutrition, Third Faculty of Medicine, Charles University in Prague, Ruská 87, Prague 10, 100 00, Czech Republic; 2 Institute of Clinical and Experimental Medicine, Vídeňská 1958/9, Prague 4, 140 21, Czech Republic

## Abstract

**Background:**

Homeless people have higher morbidity and mortality rates than the general population. Research has shown that cardiovascular disease is the leading cause of death in older homeless adults. This study was undertaken to describe the prevalence of cardiovascular risk factors in the homeless population in Prague.

**Methods:**

Data was obtained from a cross-sectional study carried out in 2003. Body mass index (BMI), waist circumference (WC), total cholesterol (TC), triacylglycerides (TAG) and smoking habits were assessed. The homeless participants in the study were recruited from a homeless center run by a Prague charitable organization called Naděje ("Hope") and at Prague's main railway station. Most participants were assessed at the Naděje center (134 persons) while the rest were assessed at Prague's Bulovka University Hospital (67 persons).

**Results:**

A total of 201 homeless (174 males and 27 females) aged 19 – 70 years were examined. Mean values of BMI, WC, TC and TAG in homeless men and women were within normal limits. Compared with the majority of the Czech population, the homeless had significantly lower mean levels of TC and TAG and lower BMI and WC values. When compared to the majority of the Czech population, the incidence of smoking among the homeless was significantly higher. Among smokers in both populations, no differences were found in the number of cigarettes smoked per day.

**Conclusion:**

Classical cardiovascular risk factors such as TC, TAG, BMI and WC, are significantly lower in Prague's homeless minority than in the majority of the Czech population. However, the prevalence of smoking is much higher in the homeless population.

## Background

Awareness of the homeless problem, in the Czech Republic, began to rise, following the overthrow of the communistic regime in 1989. It is estimated that between 35,000 to 75,000 Czechs are homeless [1]. Prague, as the capital of the Czech Republic and its most prosperous region, naturally attracts large numbers of homeless. In 2004, a field census of visible homelessness (defined by FEANTSA as rooflessness – without shelter, and houselessness – temporary institutional shelters) was carried out by Prague charitable organizations. A total of 3,096 homeless people were identified in Prague, 14% of which were women [2].

The number of homeless has increased recently. As a result, a growing body of research has focused on this disadvantaged group. Charitable organizations working with homeless people have started to investigate different aspects of the homeless lifestyle, paying particular attention to their socio-economic background and mental health. In 2004, our team published a study on the mental health of the Czech homeless population [3]. Trnka et al. performed a preventive survey regarding the prevalence of tuberculosis in the Czech homeless community [4]. However, few studies have addressed the cardiovascular health of the Czech homeless population, even though cardiovascular diseases are the leading causes of death in the Czech Republic (52.5 % in 2003) [5].

Research has shown that homeless individuals have both higher morbidity and mortality rates than the general population [6] and that cardiovascular disease is the leading cause of death in the elderly homeless [7]. Previous studies of cardiovascular risk factors in homeless populations focused either on isolated risk factors [8-12] or on providing a comprehensive risk profile [13,14]. However, these earlier studies were focused on homelessness in Western Europe or North America. Countries in those studies have different socio-economic and political backgrounds and have a much longer history in dealing with the problems of the homeless than has the Czech Republic. Our objective is to describe the prevalence of some of the major cardiovascular risk factors, namely high total cholesterol and triacylglyceride levels, smoking and obesity in those members of Prague's homeless community, that utilize local charitable organizations, and to make comparisons between this group and the general Czech population.

## Methods

### Sampling frame and subjects

A cross-sectional study of Prague's homeless population was conducted over a period of eight months in 2003. The study was carried in cooperation with Naděje ("Hope"), one of the most important day centers for homeless people in Prague. Assessments of study participants were conducted at either the Naděje Bolzanova street center, or at Prague's Bulovka University Hospital. Body mass index (BMI), waist circumference (WC), total cholesterol (TC), triacylglycerides (TAG), smoking habits and socio-demographic data were assessed.

A total of 201 homeless (86.6% men, 13.4% women), out of 217 recruited, participated in the study. Fifteen people were excluded from the study because of regular drug abuse. And one additional person was excluded because of a medical condition that precluded reliable anthropometry. Two-thirds of the homeless participants were recruited and examined at the Naděje day center (134); the remaining third of participants were recruited at Prague's main railway station (67) and examined at Prague Bulovka University Hospital. Participants in this second, smaller group were recruited with the aid of staff from Naděje and street workers at the main railway station. It was hoped that the homeless participants recruited from the railway station would represent a sample with little or no contact with charitable organizations, and as such, would provide data on those living completely unassisted. However, our assessment revealed that all recruited participants utilized Prague's charitable organizations to some extent.

Each homeless person who attended the Naděje day center was informed about the possibility of participating in the study. All those that volunteered and who met the inclusion criteria were examined. No information was gathered regarding those who did not volunteer to participate. Each study participant received a food voucher, valued at 100 CZK (approx. 3 Euros). This was a welcome incentive and much appreciated by the participants.

Each participant of the study was required to give their written consent. The study was approved by the ethical committee of the Third Faculty of Medicine, Charles University, Prague.

### Anthropometry and biochemistry

Anthropometric variables including height, weight and waist circumference were taken. Both men and women were weighed in light-weight clothing and without shoes. Measurements were taken to the nearest 0.5 kg. Weights were obtained using an electronic platform scale (SOEHNLE) with an accuracy of ± 100 g. Weight values were adjusted for clothing. Height was recorded to the nearest 0.5 cm. Height and weight values were used to determine body mass index (BMI) [kg/m^2^]. BMIs were evaluated based on the 1995 World Health Organization (WHO) interpretation [15]. Waist circumference (WC) was measured, at the narrowest part of the waist, with a plastic tape, to the nearest 0.1 cm. Abdominal obesity was defined as having a waist circumference greater than 102 cm in men and greater than 88 cm in women [16].

Venous blood samples, taken from the cubital vein, were used to determine total cholesterol (TC) and triacylglyceride (TAG) levels. All samples were tested at an accredited laboratory. Values were evaluated using Guidelines of the European Society of Cardiology 2003 [17].

### Data comparison

The homeless sample was compared with a representative sample of the general Czech population. A survey of cardiovascular risk factors in the general Czech population was conducted in 2000/2001 in nine districts of the country, involving a random sample of 1% of the population (sample age range: 25 to 64 years) [18]. For comparisons of the two populations, we used only those homeless participants that fit the age profile of the above mentioned sample – this meant using only 162 of 174 males and 24 of 27 females. Additionally, from the group that fit the age profile, some participants were eliminated prior to making educational comparisons. We decided to exclude the only male with a university education and compare only men having primary and secondary educations. A similar rational was used to exclude the two females having a secondary education. As a result, only females with primary educations were compared. Data were adjusted for age.

### Statistical analysis

Data were processed using SPSS software (Statistical Package for the Social Science) for Windows, version 12 and Epi Info, version 3.3. All group data are presented as the mean (standard deviation, SD). Data comparison was done using the t-test, χ^2 ^test, logistic regression and ACNOVA for age and sex adjustment.

## Results

The total number of homeless subjects in the study was 201. One hundred and seventy-four males, with a mean age of 42 (SD: 10.9) years (range: 19 to 70 years), and 27 females, with a mean age of 40 (SD: 9.4) years (range: 23 to 55 years). The mean period of homelessness was 2.2 years for men and 2.3 years for women. Most of the study participants (79%) were homeless for less than three years. Long-term homelessness (> 7 years) was found in only 8% of the male participants and in 11% of the female participants. Men were more educated than women: 78% of homeless men had completed secondary school, compared to only 33% of homeless women, for whom primary education was most common (63%). The majority of the participants were Czech nationals (92%). When asked about their most important food sources (using questions with multiple choice answers), most study participants reported charities (76% men, 74% women) and supermarkets (77% men, 44% women).

**Figure 1:**
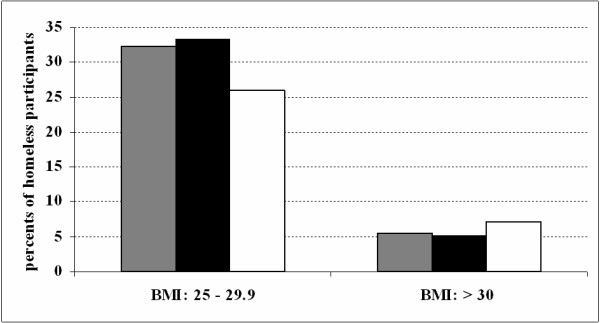
**Prevalence of overweight and obesity in the homeless population**. Grey bars: total study population (n = 201); black bars: males (n = 174); white bars: females (n = 27). Overweight: BMI 25 – 29.9 kg/m^2^; obesity: BMI > 30 kg/m^2^.

**Figure 2:**
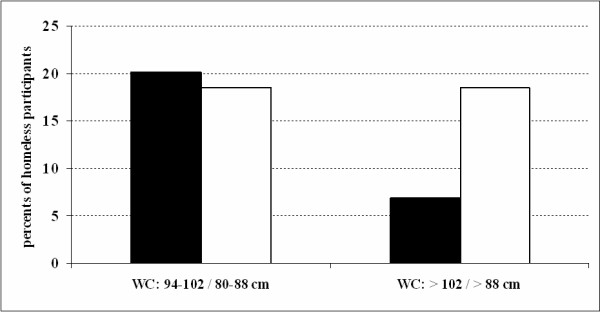
**Prevalence of increased and very high waist circumference in the homeless population**. Black bars: males (n = 174); white bars: females (n = 27). Increased waist circumference: WC 94 – 102 cm for men, 80 – 88 cm for women; very high waist circumference: WC > 102 cm for men, > 88 cm for women.

**Figure 3:**
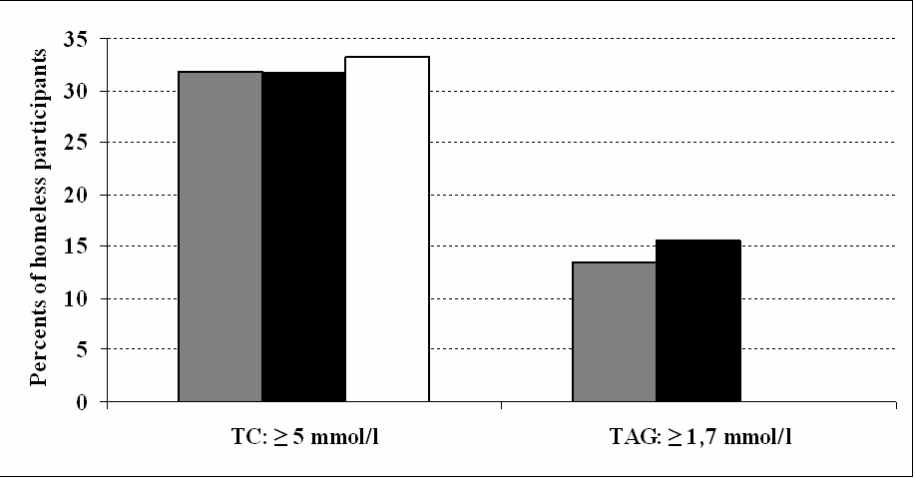
**Prevalence of increased total cholesterol and triacylglyceride level in the homeless population**. Grey bars: total study population (n = 201); black bars: males (n = 174); white bars: females (n = 27). Increased total cholesterol: TC ≥ 5 mmol/l; increased triacylglyceride level: TAG ≥ 1.7 mmol/l.

Mean values of BMI, waist circumference, total cholesterol and triacylglycerides were within normal limits (Table 1). Prevalence of being overweight and obese (BMI ≥ 25 kg/m^2^) in homeless men and women was similar (39% men, 33% women). However, obese homeless women tended to be at the higher end of the scales (Fig. 1). The prevalence of increased waist circumference (94 – 102 cm in men, 80 – 88 cm in women) and very high waist circumference (i.e. abdominal obesity, WC greater than 102 cm for men and greater than 88 cm for women) in the study participants is presented in Fig. 2. Figure 3 shows the prevalence of increased total cholesterol (> 5 mmol/l) and triacylglyceride levels (> 1.7 mmol/l). The percentage of cigarette smokers among the homeless was extremely high: 91.4% of the men and 77.8% of the women reported they smoke cigarettes. Mean lifetime exposure was 22 ± 10.4 years for men and 23 ± 7.7 years for women; men smoked, on average, 17, and women 14 cigarettes per day. A comparison of the mean values of cardiovascular risk factors including smoking, between the homeless population and the general Czech population, is presented in Table 2. The prevalence of cardiovascular risk factors is compared in Table 3. With the exception of smoking, all risk factors were significantly lower and less common in the homeless participants.

**Table 1:** Anthropological and biological indicators in the homeless sample

**Characteristic**	**Men, n = 174** mean (SD)	**Women, n = 27** mean (SD)
Waist circumference (cm)	88.0 (10.5)	78.6 (11.0)
Body mass index (kg/m^2^)	24.3 (3.9)	24.1 (5.0)
Total cholesterol (mmol/l)	4.6 (0.9)	4.8 (0.9)
Triacylglycerides (mmol/l)	1.2 (0.9)	1.0 (0.3)

**Table 2:** Comparison of mean values of the cardiovascular risk factors among the homeless and the general Czech population

**Cardiovascular risk factors in men**	**Homeless, n = 162** mean (SD)	**General population, n = 1,408** mean (SD)	**p value**
age (yrs)	43.0 (9.7)	46.5 (10.9)	< 0.001
BMI (kg/m^2^)	24.4 (3.9)	28.2 (4.4)	< 0.001
waist circumference (cm)	88.1 (10.5)	97.3 (11.9)	< 0.001
total cholesterol (mmol/l)	4.6 (0.9)	5.9 (1.1)	< 0.001
triglycerides (mmol/l)	1.2 (0.9)	2.0 (1.4)	< 0.001
length of smoking (yrs)	22.6 (9.9) (n = 140)	24.8 (10.7) (n = 539)	n.s.
number of cigarettes per day	17.3 (9.2) (n = 149)	17.2 (8.8) (n = 505)	n.s.

**Cardiovascular risk factors in women**	**Homeless, n = 24** mean (SD)	**General population, n = 543** mean (SD)	**p value**

age (yrs)	40.1 (8.8)	42.4 (8.1)	n.s.
BMI (kg/m^2^)	24.3 (5.2)	27.2 (5.6)	< 0.05
waist circumference (cm)	78.3 (11.4)	83.7 (13.0)	< 0.05
total cholesterol (mmol/l)	4.9 (0.8)	5.7 (1.1)	< 0.001
triglycerides (mmol/l)	1.0 (0.3)	1.4 (0.8)	< 0.001
length of smoking (yrs)	24.2 (6.9) (n = 17)	20.3 (8.4) (n = 192)	0.07
number of cigarettes per day	13.7 (8.1) (n = 20)	11.7 (6.4) (n = 177)	n.s.

p values hold after the adjustment for age

**Table 3:** Comparison of prevalence of the cardiovascular risk factors among the homeless and the general Czech population, (%)

**Cardiovascular risk factors**	**Homeless population**		**General Czech pop.**		**p value**
		
	men n = 162	women n = 24	men n = 1408	women n = 543	
BMI ≥ 25 kg/m^2^	41	33	75	60	< 0.001
Abdominal obesity, men (WC > 102 cm)	6		31		< 0.001*
Abdominal obesity, women (WC > 88 cm)		21		31	n.s.*
Total cholesterol ≥ 5 mmol/l	33	38	80	72	< 0.001
Triacylglycerides ≥ 1.7 mmol/l	15	0	48	23	< 0.001
Active smokers	93	83	38	35	< 0.001

p values hold after the adjustment for age and sex

*Abdominal obesity was evaluated for each sex separately because of different cut-off points.

In this case, p values are age adjusted only.

## Discussion

Cardiovascular disease is the major cause of premature death in most European populations, and it is the leading cause of death in the Czech population as well. Our study is the first one to assess cardiovascular risk factors in a sample of Prague's homeless adult population which utilize charitable organizations as part of their lifestyle. We found the prevalence of smoking to be significantly higher in homeless men (93%) and women (83%) compared to the general Czech population, men 38% and women 35%. The percentage of homeless that smoke is almost 3 times that of the general population. The homeless tended to have smoked longer; however, the number of cigarettes smoked per day didn't statistically differ from the figure for the general population. A high prevalence of smoking in homeless populations has also been found by other authors [11,13,14,19,20], however none of the reported levels were as extreme as what we found in our study participants. Smoking is associated with a lower socioeconomic status [21] and a lower degree of education [22]. Additionally, the prevalence of nicotine dependence among alcohol or other substance abusers is extremely high [23] and the high degree of alcohol and drug use/abuse among the homeless is well documented [11,24,25]. The higher incidence of smoking in our study participants may be attributable to stress induced by the homeless lifestyle or substance abuse. Additionally, many homeless engage in alternative smoking behaviors, such as smoking discarded cigarette butts and used filters that increase the potential for toxin and infectious agent intake [26].

Hypercholesterolemia and hypertriglyceridemia were significantly lower in the homeless compared to the majority of the Czech population. This may be a result of decreased accessibility to food, and/or (somewhat more questionable), higher physical activity levels (associated with the homeless lifestyle) compared to the general population. However, an earlier study by Luder et al. [27] showed a high prevalence of hypercholesterolemia (92% above 5.17 mmol/l) and also high intakes of saturated fat and cholesterol among the homeless in New York City, however, this might be due to a different diet profile. Unfortunately, dietary intake was not a subject of our study, and therefore its role in our participants remains speculative. Further research needs to be done to investigate this connection.

The homeless were significantly less likely to be overweight or obese (BMI ≥ 25 kg/m^2^) than the general Czech population. Our results document a lower prevalence of overweight and obesity than previous studies. Lee et al. found 46% of Toronto's homeless adults to be either overweight or obese [14]. An even greater prevalence (54.5%) was documented by Luder et al. [27]. The prevalence of obesity in homeless men and women was similar; however, obese homeless women tended to have a higher degree of obesity and also a higher prevalence of abdominal obesity. This could be related to the fact that mental problems (depressions and anxiety) are more frequent in women than in men [3], which is often coupled with higher alcohol consumption and its concomitant caloric intake.

In addition, waist circumference was assessed as an indicator of abdominal obesity and as such, a risk factor for cardiovascular diseases. The prevalence of abdominal obesity was much lower in the homeless than in the general population. It has been shown that abdominal obesity is positively correlated with the intake of total fat [28] and smoking, and negatively correlated with protein intake [29]. A nutritional survey of the daily consumption of the homeless would go a long way towards verifying this assumption.

## Conclusion

We conclude that the Prague homeless population (at least those meeting our inclusionary criteria) has a higher prevalence of smoking than the general Czech population. On the other hand, their lifestyle appears to result in lower BMIs, waist circumference, total cholesterolaemia and triglyceridaemia. It is not certain whether these results can be generalized to the entire homeless population. The participants in our study group consisted of homeless utilizing the services of charitable organizations, and, therefore, may not fully represent the entire homeless population. However, it is this particular subgroup of the homeless that is most likely to benefit from preventive measures and interventions, and for this reason, it represents an important subpopulation of the homeless community.

To reduce smoking in the homeless community will require intervention. However, any truly effective intervention program will require political and socioeconomic changes.

## Competing interests

The author(s) declare that they have no competing interests.

## Authors' contributions

DK coordinated the study, supervised the research group, contributed to the study design, data collection, analysis and interpretation, and helped to draft the manuscript. VL participated in statistical analysis, especially with the data comparisons and made essential contributions to data interpretation. VA made a substantial contribution to data acquisition and participated in the statistical analysis. PD participated in the conception and design of the study, data interpretation, and drafting of the manuscript. JR participated in data collection, and assisted in drafting the manuscript. MA participated in the conception of the study, and data interpretation. All authors read and approved the final manuscript.

## Pre-publication history

The pre-publication history for this paper can be accessed here:


